# Metal Atom Effect on the Photophysical Properties of Mg(II), Zn(II), Cd(II), and Pd(II) Tetraphenylporphyrin Complexes Proposed as Possible Drugs in Photodynamic Therapy

**DOI:** 10.3390/molecules22071093

**Published:** 2017-06-30

**Authors:** Bruna Clara De Simone, Gloria Mazzone, Nino Russo, Emilia Sicilia, Marirosa Toscano

**Affiliations:** Dipartimento di Chimica e Tecnologie Chimiche, Università della Calabria, I-87036 Arcavacata di Rende, Italy; bruna.desimone@unical.it (B.C.D.S.); gloria.mazzone@unical.it (G.M.); siciliae@unical.it (E.S.); marirosa.toscano@unical.it (M.T.)

**Keywords:** spin-orbit matrix elements, absorption spectra, singlet-triplet energy gaps, TDDFT, tetraphenylporphyrin complexes, PDT

## Abstract

The effects of Mg, Zn, Cd, and Pd dications on the photophysical properties of the tetraphenylporphyrin ligand have been explored, considering the corresponding complexes and by using the density functional theory and its time-dependent extension. Results show that absorption wavelengths do not change significantly when the metal ion changes contrary to what happens to the singlet–triplet energy gaps (ΔE_S−T_) and the spin-orbit matrix elements $\langle \Psi_{S_{n}}|{\hat{H}}_{\mathrm{so}}{|\Psi }_{T_{m}}\rangle$. The most probable intersystem spin crossing (ISC) pathways for the population of the lowest triplet states have been explored. Our findings can contribute to rationalize the available experimental data and promote the potential therapeutic use of these compounds as photosensitizers in photodynamic therapy (PDT).

## 1. Introduction

The transitions between pure spin states of different multiplicities are, as it is well known, forbidden by the spin selection rules. However, several spin-forbidden intersystem crossings in organic and inorganic systems are reported to be essential for a specific action in different areas [1,2,3]. It is known that these transitions can occur as result of a spin–orbit coupling (SOC), a relativistic effect that induces a quantum mechanical mixing between states with different spin multiplicity. The SOC arises from the interaction between spin magnetic moment of an electron and the magnetic field resulting from the motion of the nucleus. Since the nuclear magnetic field depends on nuclear charge, the SOCs assume greater values with the increasing of the atomic number. High SOC values enhance the kinetics of both radiative and non-radiative transitions between states with different spin. Based on what has been said before, the intersystem crossing (ISC) is observed often in systems containing high atomic number elements. The phenomenon is, thus, known as heavy atom effect (HAE).

HAE plays an important role in determining many photophysical and photochemical properties. The phosphorescence lifetime of various substituted aromatic compounds has been associated with the spin–orbit coupling variation due to the presence of different heavy atoms in the examined species, for the first time by McClure’s [4]. Later, the HAE in photophysics and photochemistry has assumed increasing importance [1,5,6]. Nowadays, a great deal of interest in the ISC process is related to design of novel materials useful in many fields such as photocatalytic organic and inorganic reactions, triplet-triplet annihilation, and photodynamic therapy (PDT).

PDT is a clinical therapeutic modality with great potential of application in various diseases, including cancer photo-treatment [7,8,9,10,11,12,13], but also in environmental and antimicrobial fields [14,15,16,17]. PDT combines the action of three key components: (i) the light, with specific wavelength; (ii) a photosensitizer; and (iii) molecular oxygen naturally present in the tissues, in order to produce reactive oxygen species, like singlet excited oxygen, as cytotoxic agents that are able to destroy diseased cells selectively. The success of this strategy lies in an effective ISC between singlet and triplet excited states of the employed photosensitizer. For this reason, the research on design and characterization of new drugs for PDT is a matter of great interest. An ideal PDT photosensitizer must possess, besides certain chemical properties, several photophysical properties. Among them, the most important are: (i) the absorption in the so-called photodynamic therapeutic window (500–900 nm) in order to maximize the penetration into the tissues; (ii) an efficient intersystem spin crossing to populate a triplet state (S_n_ → T_m_); (iii) a subsequent accessible triplet state energy transfer needed to excite the molecular oxygen from its ^3^Σ_g_ ground state to the excited one ^1^Δ_g_. This means that the ΔE_S−T_ of the photosensitizer must be higher than 0.98 eV, the energy required to produce the cytotoxic agent (^1^O_2_) in type II photoreactions [18].

Several classes of compounds have been studied so far and the porphyrin-like systems (e.g., foscan, temocene, and their metal complexes) still remain one of the most interesting families [19,20,21,22,23,24,25]. The presence of a metal in the porphyrin-like cavity should, in principle, increase the ISC kinetics due the presence of a coordinated metal (internal heavy atom effect).

**Scheme 1:**
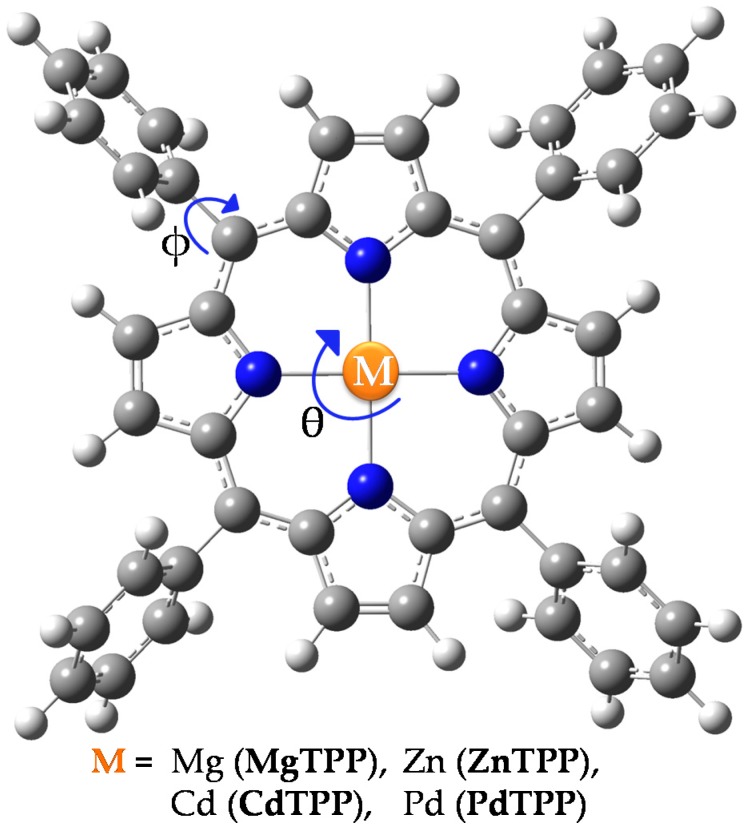
Representation of the investigated metallotetraphenylporphyrines (MTPP, with M = Mg(II), Zn(II), Cd(II), Pd(II)).

In this work, we have undertaken a systematic theoretical work, in the framework of Density Functional Theory (DFT) and its time-dependent extension (TDDFT), on the photophysical properties of Mg(II), Zn(II), Cd(II), and Pd(II) tetraphenylporphyrin (TPP) complexes (see Scheme 1). In particular, we have tried to elucidate the origin of the improved ^1^Δ_g_ production besides the excitation energies, the singlet-triplet energy gaps, the spin-orbit matrix elements, directly related to the intersystem crossing kinetics as postulated by the Fermi Golden Rule [26,27]. Moreover, different possible intersystem spin crossing pathways for the activation of the lowest triplet state have been explored and discussed on the basis of the obtained SOC and ΔE_S-T_ values taking into account the El-Sayed [28] and Kasha [29] rules.

## 2. Results and Discussion

Although optimizations have been carried out without imposing any constraints, resulting structures show *D_2d_* geometries with a very slight deviation from planarity around the porphyrinoid core which occurs mainly for the triplets. The maximum deviation is found for the zinc complex in the T_2_ excited state where θ has a value of 3.5°. The *meso* phenyl substituents lie out from the porphyrin ring plane in both ground and excited states of all the considered systems. The torsional angle (φ) is about 60° in the ground states and a variation of few degrees have been registered in the excited ones. Comparison with the experimental data is possible only for the zinc complex [30]. We find a Zn–N distance of 2.029 Å in good agreement with the X-ray counterpart [30] (2.037 Å). The agreement is also satisfactory with previous density functional studies in which different exchange-correlation functionals have been used [31,32]. As expected, in the excited states other geometrical parameters also change, but the variations are very small.

The computed excitation energies together with the available experimental data are collected in Table 1.

The analysis of the molecular orbitals energies has evidenced that HOMO and HOMO − 1, as well as LUMO and LUMO + 1, are doubly degenerate, in agreement with previous studies [31,32]. This means that the excited states generated by transitions involving degenerate orbitals result to be equally degenerate, such as S_1_ and S’_1_ deriving from H → L and H → L + 1 transitions (see Table 1), respectively. Therefore, only one value will be used for the discussion.

As in the case of other porphyrin-like metal complexes [31], the *D_2d_* molecular symmetry of metallotetraphenylporphyrin complexes MTPP is responsible of the single Q and Soret bands, generally split in two different bands in metal-free tetraphenylporphyrin. As usual, the Q band has a very low oscillator strength, while the Soret one is the most intense band. Both the singlet transitions are in satisfactory agreement with the experimental data [33,34,35], being the average error about 40 and 20 nm, respectively. The maximum deviation occurs for the Q band of **CdTPP** for which a difference from the experiment value of 68 nm has been found. The degenerate S_1_ and S’_1_ excited states, that fall in the Q region, are mainly originated by a H → L and H → L + 1 transitions, respectively, while the Soret band, with two degenerate states, is obtained by H – 1 → L (S_2_) and H − 1 → L + 1 S’_2_ transitions. The comparison of our results with the available experimental data concerning the lowest triplet excitation energies for Mg, Zn, and Cd-complexes [35] shows an excellent agreement (average error of 0.06 eV). On the basis of this evidence, we think that our prediction on the lowest triplet excitation energy for **PdTPP** complex should be reliable.

From the computed vertical transition energies that emerge in all the studied systems, the energy gap between the ground singlet and low lying triplet excited states (${\Delta E}_{S_{0}-T_{1}}$) is higher than the energy required to excite the molecular oxygen in its ^1^Δ_g_ state (0.98 eV) and, consequently they are potential candidates to act as type II photosensitizers in PDT.

The production of cytotoxic singlet oxygen is directly related to the rate constant of the radiationless intersystem crossing (ISC) between S_n_ and T_m_ states. In the golden rule approximation [26,27], the ISC efficiency depends on the amplitude of the spin−orbit coupling and the related $\Delta E_{S_{n}-T_{m}}$ energy gaps. The first term is proportional to the squared module of the spin−orbit matrix element between the initial Ψ(S_n_) and final Ψ(T_m_) wave functions that depends on the nature of the orbitals involved in the transition (according to the El Sayed rules [28]). Heavy atoms can also affect the SOCs that increase as a function of Z^4^ (heavy atom effect) being directly proportional to the atomic number and inversely proportional to the mean cubic radial distribution (r^−3^) of the electron. 

**Scheme 2:**
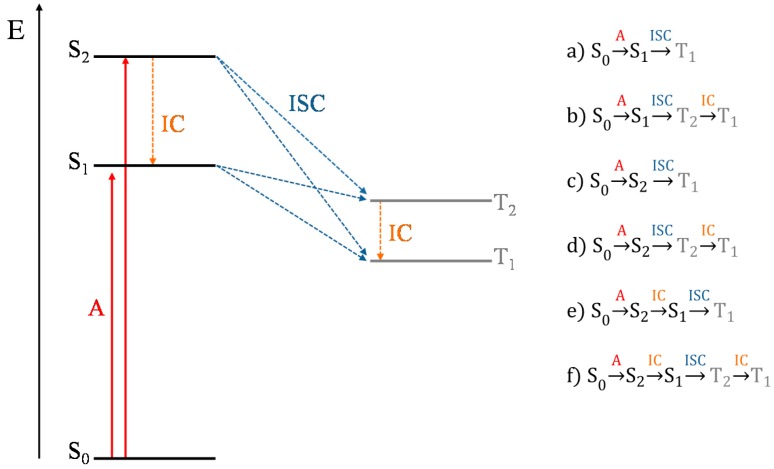
Representative energy diagram of the excited singlet and triplet states and likely deactivation pathways (**a**–**f**).

Given the presence of two low-lying singlet excited states and the existence of triplets below them, all the deactivation processes for the production of singlet oxygen depicted in Scheme 2 have been considered. In these paths, the deactivation can start from: (i) the first excited singlet state S_1_ generated by a transition with very weak oscillator strength, or (ii) the second excited singlet state S_2_ that can directly decay on the triplets or through a fast S_2_ → S_1_ internal conversion. The available experimental data provide controversial interpretations about the origin of the emission for these systems [33,34,35,36,37,38,39]. In some studies, the emission band is interpreted as coming directly from S_2_, whereas others suggest that the S_1_ state is involved, assuming that an IC from S_2_ to S_1_ occurs. Although the first hypothesis, as mentioned above, is the most accredited we have considered the possible deactivation pathways taking into account both S_1_ and S_2_ excited states as the starting state for the population of the lowest triplet one. Therefore, in order to suggest the preferred deactivation path, the following SOCs have been computed: $\langle \Psi_{S_{1}}{|\hat{H}}_{\mathrm{so}}{|\Psi }_{T_{1}}\rangle$, $\langle \Psi_{S_{1}}{|\hat{H}}_{\mathrm{so}}{|\Psi }_{T_{2}}\rangle$, $\langle \Psi_{S_{2}}{|\hat{H}}_{\mathrm{so}}{|\Psi }_{T_{1}}\rangle$ and $\langle \Psi_{S_{2}}|{\hat{H}}_{so}|\Psi_{T_{2}}\rangle$. Table 2 reports these values together with the relative ${\Delta E}_{S_{n}-T_{m}}$ values.

In the first pathway (a), the T_1_ state can be populated by a direct ISC from S_1_ state while in the second (b) the S_1_ → T_2_ ISC is followed by a fast IC to the lowest triplet state T_1_. For all the MTPP systems the $\langle \Psi_{S_{1}}{|\hat{H}}_{\mathrm{so}}{|\Psi }_{T_{1}}\rangle$ term assumes the greatest value and the energy gap between the involved excited states, $\Delta E_{S_{1}-T_{1}}$, is about 0.7 eV. The decay of S_2_ to triplets T_1_ and T_2_ is taken into account in paths (**c**) and (**d**), respectively. From the computed SOCs and singlet–triplet energy gaps (see Table 2) it seems that the path (**d**), involving S_2_ → T_2_ radiationless transition, is the preferred one. However, the SOC values end up being smaller than those found for the ISC involving the S_1_ state. In the hypothesized deactivation (**e**) and (**f**) channels, a primary IC from S_2_ to S_1_ is followed by the ISC to the T_1_ or T_2_ states, respectively. As just mentioned, the obtained $\langle \Psi_{S_{1}}{|\hat{H}}_{\mathrm{so}}{|\Psi }_{T_{1}}\rangle$ values, being approximately twice than those computed for the S_1_ → T_2_ transition, suggest the pathway (**e**) as the most probable one. This is in agreement with the El Sayed [28] and Kasha [29] rules.

**Figure 1:**
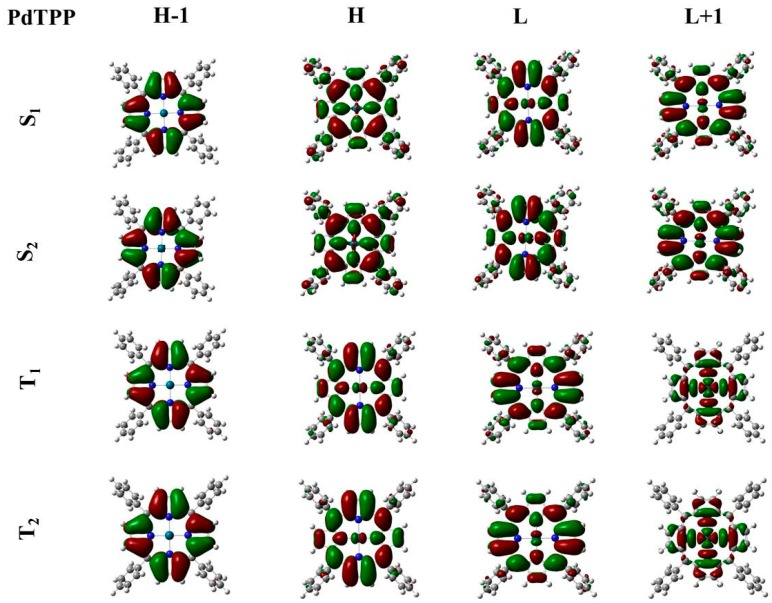
Graphical representation of the HOMO − 1, HOMO, LUMO and LUMO + 1 for S_1_, S_2_, T_1_, T_2_ states of **PdTPP** computed at M06/6 − 31G * level of theory.

From Table 2, it clearly appears how the nature of the central metal ion affects the spin–orbit coupling amplitude. As expected, the largest SOC values are obtained for **PdTPP**. The radiationless process involves excited states that are mainly π–π* in nature, with a modest participation of d metal orbitals that becomes consistent in the T_1_ and T_2_ states (see Figure 1).

**Figure 2:**
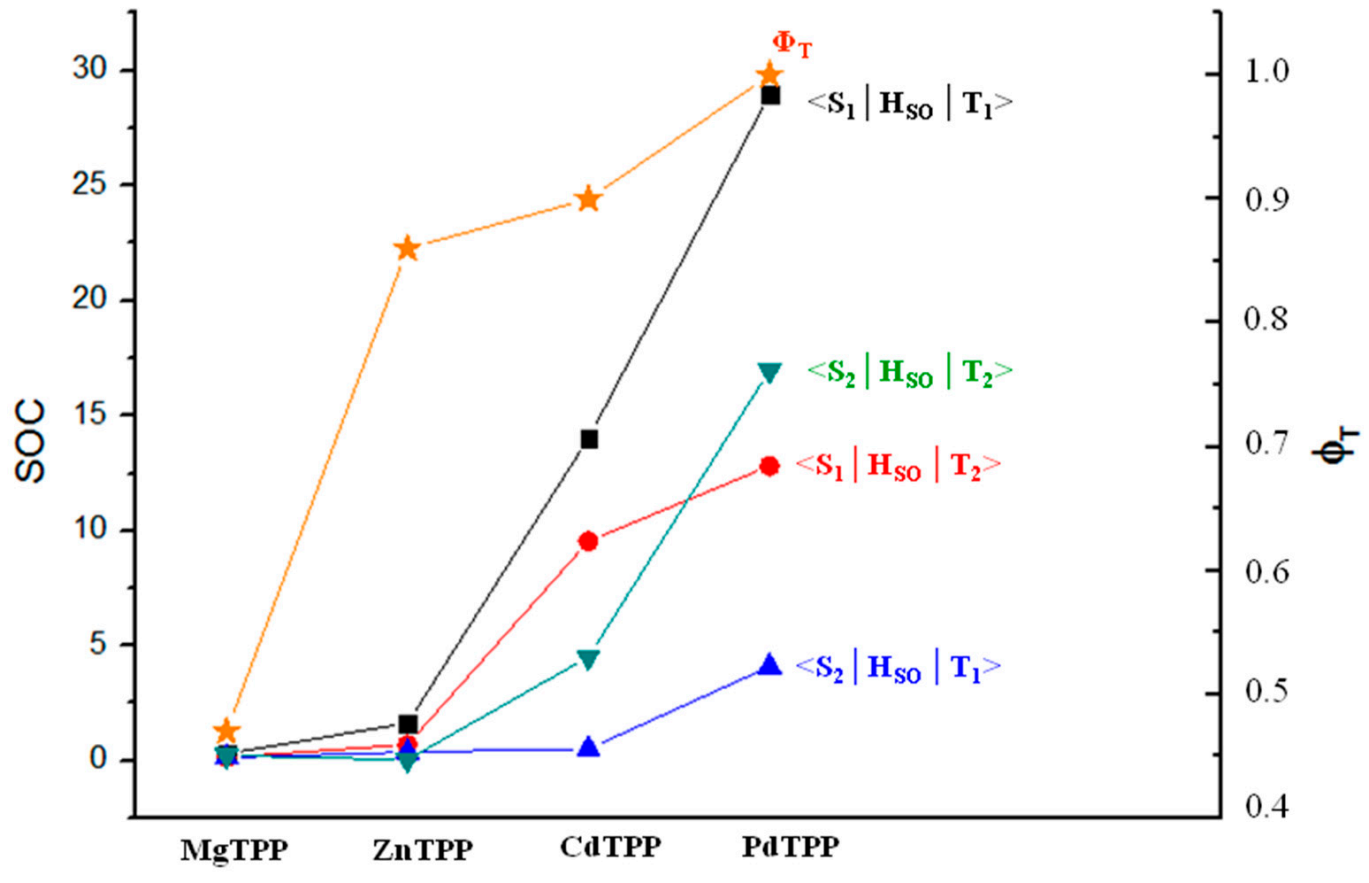
Computed SOCs (cm^-1^) for S_n_–T_m_ (with n and m = 1, 2) radiationless transitions and measured [35] triplet quantum yields ϕ_T_ for the investigated systems.

In order to compare our results with the available experimental ones, we have reported, in Figure 2, the trend of both the experimentally determined triplet quantum yields (ϕ_T_) [35] and our SOC values. As shown in figure, the increase of ϕ_T_ as a function of metal atom size is well reproduced by the trend of $\langle \Psi_{S_{1}}{\hat{H}}_{\mathrm{so}}{|\Psi }_{T_{1}}\rangle$ along the series, especially in the cases of **MgTPP** and **PdTPP**.

Although deactivation pathways that involve the ISC between S_1_ and T_1_ seem to be the main events, other paths can contribute to the production of singlet oxygen since all the computed SOCs are higher than that computed for Foscan^®^ (2.4 × 10^-1^ cm^-1^ for the S_1_ → T_1_ transition) [22], the photosensitizer already used in photodynamic therapy.

## 3. Computational Methods

Ground and excited state optimizations have been performed by using M06 [40] exchange correlation functional coupled with the all electron 6-31G* basis sets for all the atoms except for Cd and Pd, for which the SSD pseudopotential has been employed for DFT and TDDFT levels of theory. DFT and TDDFT approaches have been successfully applied to study the photophysical properties of similar and other systems [22,23,24,25,41,42,43,44,45,46,47].

The excitation energies have been calculated by substituting the 6 − 31G * basis set with the 6 − 31 + G* one. Solvent effects have been included in the excitation energy computations by means of integral equation formalism polarizable continuum model (IEFPCM) [48,49]. Since the experimental data [33,34,35] have been obtained in benzene solution, we have considered this solvent (ε = 2.27). All these computations have been carried out by using Gaussian 09 code [50].

SOC elements have been computed with DALTON code [51] by using the spin–orbit coupling operators for effective core potentials with the effective nuclear charge [52] for systems containing Cd and Pd atoms, while atomic-mean field approximation [53] is used in the other cases. Due to the limited number of functionals implemented in this code for quadratic response calculations, the B3LYP [54,55] has been employed. The cc-pVDZ basis set has been used for all the atoms with the exception of Cd and Pd, which we have described using the cc-pVDZ-PP one.

The spin–orbit couplings (SOCs) have been defined according to the following formula

(1) $${\mathrm{SOC}}_{\mathrm{ij}}=\sqrt{\underset{n}{\sum }{\left|\langle \psi_{S_{i}}\left|{\hat{H}}_{\mathrm{SO}}\right|\psi_{T_{j,n}}\rangle \rangle \right|}^{2}};\;n=x,y,z$$

where ${\hat{H}}_{\mathrm{SO}}$ is the spin–orbit Hamiltonian.

## 4. Conclusions

Metal atom effect on the photophysical properties of metals containing tetraphenylporphyrin has been determined by using DFT and TDDFT theoretical based methods. Our results show that the employed computational protocol, that is M06/6 − 31 + G *, is able to reasonably reproduce the experimental absorption wavelengths with errors of a few tens of nm. Our computations have confirmed that the Q band lies over 550 nm, falling in the low region of the so-called therapeutic window. The evaluation of the low-lying triplet states has evidenced the possibility of all considered MTPP to be able to generate the cytotoxic agent in type II PDT photoreactions, as ΔE_S−T_ gaps are greater than the energy required to excite the molecular oxygen (experimental value 0.98 eV).

Moreover, the introduction of the metal ion in the cavity of porphyrin-like moiety and its nature induce an increase of the spin−orbit matrix elements proportionally with the increase of the atomic number. Following the El Sayed rules, the most efficient intersystem crossing occurs for **PdTPP** in which the transition between two states with different spin multiplicities involves orbitals with different shapes.

Even if all the investigated systems own photophysical properties that make them suitable to be used as photosensitizers in photodynamic therapy, we suggest the magnesium and palladium containing compounds as the best PS candidates in PDT. We hope that our investigation can stimulate further experimental work on these promising photosensitizers.

## Figures and Tables

**Table 1:** Main vertical excitation energies (ΔE), absorption wavelengths (λ) Oscillator Strengths (*f*) and Transitions (Molecular Orbital contribution in %) for the studied compounds in benzene solvent, computed at the M06/6 − 31 + G * level of theory.

Compound	Excited State	λ_theo_ (nm)	ΔE (eV)	*f*	λ_exp_ (nm)	Transitions ^d^
MgTPP	S_1_ (S’_1_)	559	2.22	0.008	607 ^a^	H → L, 54% (H → L + 1, 54%)
	S_2_ (S’_2_)	404	3.07	1.739	428 ^a^	H − 1 → L, 44% (H − 1 → L + 1, 44%)
	T_1_ (T’_1_)	831	1.49	0	794 ^c^	H → L, 83% (H → L + 1, 83%)
	T_2_ (T’_2_)	630	1.97	0		H − 1→L, 84% (H − 1 → L + 1, 84%)
ZnTPP	S_1_ (S’_1_)	561	2.21	0.038	593 ^a^	H → L, 50% (H → L + 1, 50%)
	S_2_ (S’_2_)	412	3.01	1.699	424 ^a^	H − 1 → L + 1, 47% (H − 1 → L, 47%)
	T_1_ (T’_1_)	812	1.53	0	778 ^c^	H → L, 70% (H → L + 1, 70%)
	T_2_ (T’_2_)	626	1.98	0		H − 1 → L, 82% (H − 1 → L + 1, 82%)
CdTPP	S_1_ (S’_1_)	549	2.26	0.013	617 ^a^	H → L, 54% (H → L + 1, 54%)
	S_2_ (S’_2_)	397	3.12	1.670	437 ^a^	H − 1 → L+1, 59% (H − 1 → L, 59%)
	T_1_ (T’_1_)	846	1.46	0	814 ^c^	H → L, 52% (H → L + 1, 52%)
	T_2_ (T’_2_)	619	2.00	0		H − 1 → L, 85% (H – 1 → L + 1, 85%)
PdTPP	S_1_ (S’_1_)	537	2.31	0.032	553 ^b^	H → L, 48% (H → L + 1, 48%)
	S_2_ (S’_2_)	409	3.03	1.519	407 ^b^	H − 1 → L, 59% (H − 1 → L + 1, 59%)
	T_1_ (T’_1_)	758	1.63	0		H → L, 78% (H → L + 1, 78%)
	T_2_ (T’_2_)	597	2.07	0		H − 1 → L, 84% ( H − 1 → L + 1, 84%)

^a^ [33]; ^b^ [34] in CHCl_3_; ^c^ [35]; ^d^ only the major contribution is reported.

**Table 2:** SOC values (cm^-1^) and energy gaps (${\Delta E}_{S_{n}-T_{m}}$ eV) between the involved excited states calculated at B3LYP/cc-pVDZ//M06/6 − 31G * and M06/6 − 31 + G* levels of theory.

	MgTPP	ZnTPP	CdTPP	PdTPP
$\left|\langle \Psi_{S_{1}}|{\hat{H}}_{so}|\Psi_{T_{1}}\rangle \right|$ $\Delta E_{S_{1}-T_{1}}$	0.31 0.73	1.64 0.68	14.01 0.80	28.99 0.68
$\left|\langle \Psi_{S_{1}}|{\hat{H}}_{so}|\Psi_{T_{2}}\rangle \right|$ $\Delta E_{S_{1}-T_{2}}$	0.15 0.25	0.70 0.23	9.55 0.26	12.81 0.24
$\left|\langle \Psi_{S_{2}}|{\hat{H}}_{so}|\Psi_{T_{1}}\rangle \right|$ $\Delta E_{S_{2}-T_{1}}$	0.14 1.58	0.38 1.48	0.52 1.66	4.10 1.40
$\left|\langle \Psi_{S_{2}}|{\hat{H}}_{so}|\Psi_{T_{2}}\rangle \right|$ $\Delta E_{S_{2}-T_{2}}$	0.22 1.10	0.05 1.03	4.52 1.12	16.97 0.96
